# The Fungal Metabolite Eurochevalierine, a Sequiterpene Alkaloid, Displays Anti-Cancer Properties through Selective Sirtuin 1/2 Inhibition

**DOI:** 10.3390/molecules23020333

**Published:** 2018-02-05

**Authors:** Michael Schnekenburger, Véronique Mathieu, Florence Lefranc, Jun Young Jang, Marco Masi, Anake Kijjoa, Antonio Evidente, Hyun-Jung Kim, Robert Kiss, Mario Dicato, Byung Woo Han, Marc Diederich

**Affiliations:** 1 Laboratoire de Biologie Moléculaire et Cellulaire du Cancer, Hôpital Kirchberg, 9, rue Edward Steichen, L-2540 Luxembourg, Luxembourg; michael.schnekenburger@lbmcc.lu (M.S.); dicato.mario@chl.lu (M.D.); 2 Department of Pharmacotherapy and Pharmaceutics, Faculté de Pharmacie, Université Libre de Bruxelles, Boulevard du Triomphe, 1050 Brussels, Belgium; vemathie@ulb.ac.be; 3 Service de Neurochirurgie, Hôpital Erasme, Université Libre de Bruxelles, 808 Route de Lennik, 1070 Brussels, Belgium; florence.lefranc@erasme.ulb.ac.be; 4 Department of Pharmacy, Research Institute of Pharmaceutical Sciences, College of Pharmacy, Seoul National University, 1 Gwanak-ro Gwanak-gu, Seoul 08826, Korea; nosvc4@snu.ac.kr; 5 Dipartimento di Scienze Chimiche, Università di Napoli Federico II, Via Cintia 4, 80126 Napoli, Italy; marco.masi@unina.it (M.M); evidente@unina.it (A.E.); 6 Instituto de Ciências Biomédicas de Abel Salazar (ICBAS) and CIIMAR, Universidade do Porto, Rua de Jorge Viterbo Ferreira, 228, 4050-313 Porto, Portugal; ankijjoa@icbas.up.pt; 7 College of Pharmacy, Chung-Ang University, 84 Heukseok-ro Dongjak-gu, Seoul 06974, Korea; hyunjungkim@cau.ac.kr; 8 Retired—previously Director of Research at the Fonds National de la Recherche Scientifique (FRS-FNRS; Belgium); rkiss2012@gmail.com

**Keywords:** cancer, epigenetics, HDAC, sirtuin inhibitor, natural compound, eurochevalierine, cytostatic compound

## Abstract

NAD^+^-dependent histone deacetylases (sirtuins) are implicated in cellular processes such as proliferation, DNA repair, and apoptosis by regulating gene expression and the functions of numerous proteins. Due to their key role in cells, the discovery of small molecule sirtuin modulators has been of significant interest for diverse therapeutic applications. In particular, it has been shown that inhibition of sirtuin 1 and 2 activities is beneficial for cancer treatment. Here, we demonstrate that the fungal metabolite eurochevalierine from the fungus *Neosartorya pseudofischeri* inhibits sirtuin 1 and 2 activities (IC_50_ about 10 µM) without affecting sirtuin 3 activity. The binding modes of the eurochevalierine for sirtuin 1 and 2 have been identified through computational docking analyses. Accordingly, this sequiterpene alkaloid induces histone H4 and α-tubulin acetylation in various cancer cell models in which it induces strong cytostatic effects without affecting significantly the viability of healthy PBMCs. Importantly, eurochevalierine targets preferentially cancer cell proliferation (selectivity factor ≫ 7), as normal human primary CD34^+^ stem/progenitor cells were less affected by the treatment. Finally, eurochevalierine displays suitable drug-likeness parameters and therefore represent a promising scaffold for lead molecule optimization to study the mechanism and biological roles of sirtuins and potentially a basis for development into therapeutics.

## 1. Introduction

The European Union [1] and the United States of America (USA) [2] as well as most developed countries are facing an increasing health problem with respect to cancer. In the European Union, cancer represents the second most important cause of death and morbidity with one out of four deaths [1]. In the USA, about 1.7 millions new cancer cases and about 610,000 cancer deaths are projected for 2018 [2]. 

There is increasing evidence that, besides genetic lesions, epigenetic alterations including DNA methylation, small non-coding RNAs, and histone modifications are regulating essential steps of carcinogenesis [3,4,5,6]. Among the enzymes responsible for regulating epigenetic marks, histone deacetylases (HDACs), the enzyme responsible for the removal of lysine acetyl groups, became an interesting target for anti-cancer therapy [7,8]. There are 18 members in the HDAC family classified into four major groups. Class I, II, and IV HDACs are zinc-dependent, whereas Class III HDACs, also called sirtuins (SIRTs), require nicotinamide adenine dinucleotide (NAD^+^) as a co-factor [9,10]. Sirtuins (SIRT1-7) share extensive homologies with the yeast HDAC Silent Information Regulator 2 (Sir2). SIRT1 is essentially nuclear and modulates acetylation of histone H1, H3, and H4, whereas SIRT2 is predominantly cytoplasmic and regulates α-tubulin, together with HDAC6 [11]. HDAC inhibitors (HDACi) of class I, II, and IV are the most widely used for basic research as well as in the clinic [12,13]; however, sirtuin inhibitors (SIRTi) represent a novel, attractive approach for anti-cancer drug development [6,7]. Hence, numerous reports demonstrated that SIRT1, SIRT2, or dual SIRT1/2 inhibitors may generate anticancer effects. However, depending on the inhibitors and cancer cell models, the observed antitumor effects and cellular outcomes are not always comparable and can be mediated by various mechanisms [7]. For instance, the SIRT1/2 inhibitor sirtinol triggers growth arrest in human breast and lung cancer cells [14]. Similarly, the benzopyran-based compound 18 inhibits SIRT1/2 activities and acts as a cytostatic agent in glioblastoma cells [15], whereas the dual SIRT1/2 inhibitor tenovin-1 induces apoptosis in sarcoma cells [16]. Other specific sirtuin inhibitors (SIRTi) have been reported to trigger cancer cell death, including the SIRT1 inhibitors NCO-01 and -04, and the SIRT2 inhibitors AEM1 and AEM2, which induce cell death in T-cell leukemia/lymphoma [17] and in non-small cell lung cancer [18], respectively. Nevertheless, several reports have demonstrated that the knockdown of SIRT1, SIRT2, or SIRT1/2 genes affected less cancer cell viability but essentially proliferation and/or invasion [19].

**Figure 1:**
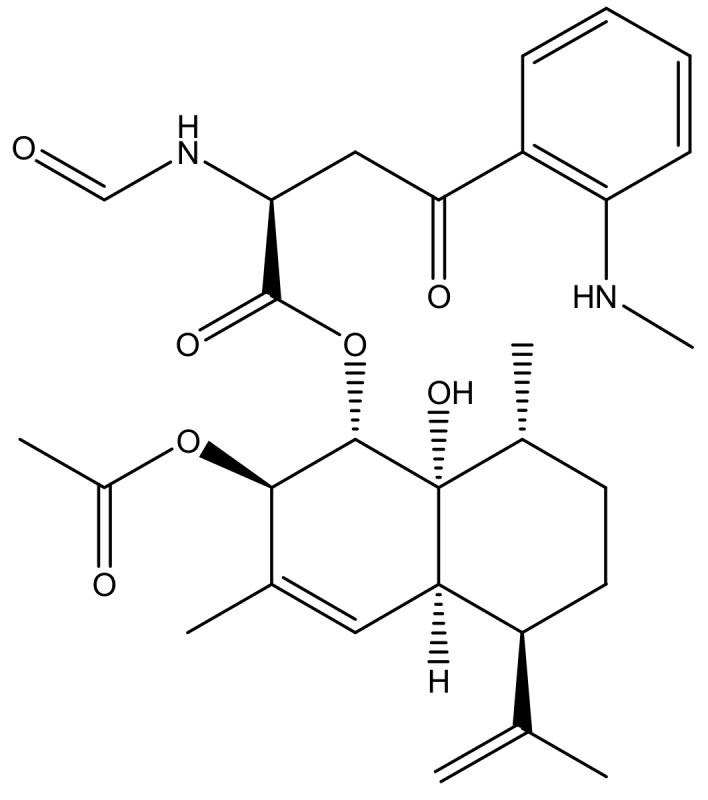
Structure of eurochevalierine.

Natural products and their semisynthetic or synthetic derivatives represent more than 50% of the current armamentarium to combat cancer [20,21,22,23,24,25]. Various natural compounds display anti-HDAC activity [8,10,26] and the current study details the anti-HDAC activity of eurochevalierine, a sequiterpene alkaloid (Figure 1), which is a fungal metabolite isolated from *Eurotium chevalieri* Mangin that is an imperfect stage of *Aspergillus chevalieri* (Mangin) Thorn and Church and that belongs to Phylum *Ascomyceta* [27]. We previously showed that eurochevalierine is a cytostatic but not cytotoxic compound in vitro with respect to the growth of various cancer cell models [28]. Eurochevalierine does not induce apoptosis in cancer cells and displays its cytostatic effects even in apoptosis-resistant cancer cells [28].

The aim of this work was to further investigate epigenetic regulatory mechanisms that could account for the observed inhibition of cancer cell proliferation. Based on computational docking experiments combined with in vitro HDAC activity assays and the examination of the profile of acetylation of α-tubulin and several histone marks in various solid cancer models, we established that eurochevalierine leads to specific inhibition of SIRT1 and 2.

## 2. Results

**Figure 2:**
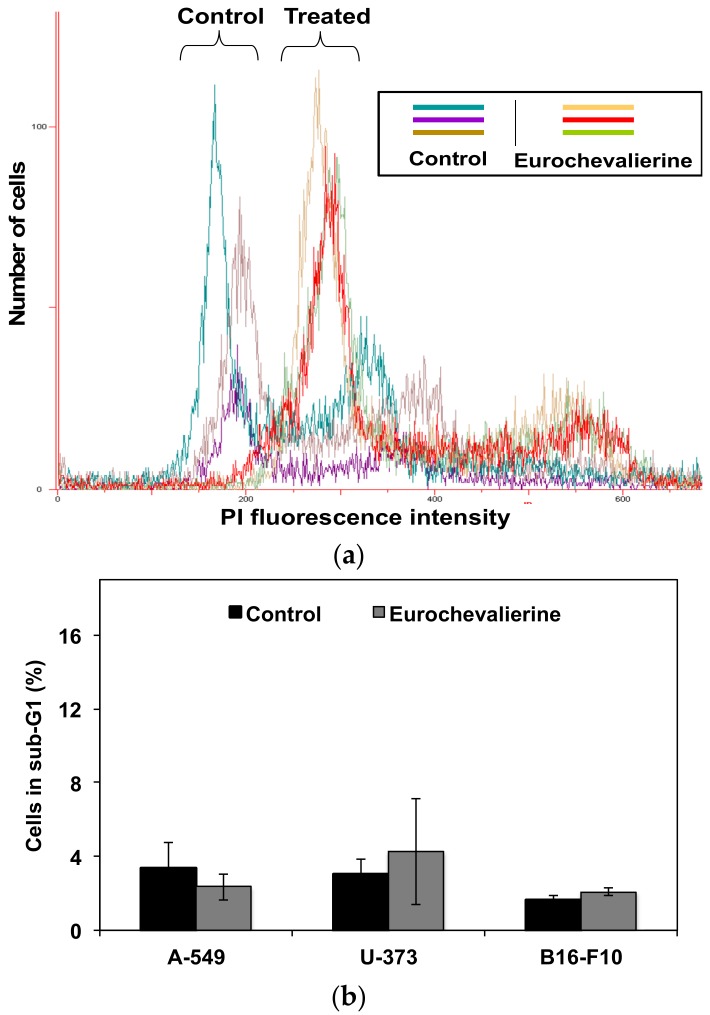
Effect of eurochevalierine on cell cycle distribution. Cells were treated with 50 µM eurochevalierine for 72 h and then stained with propidium iodide (PI), and DNA content was measured by flow cytometry. (**a**) Cell cycle distribution profile in A-549 cells. Results of three independent experiments are represented on the same graph. (**b**) Quantification of the number of cells in sub-G1 fraction as a percentage of the total cell population. Results are expressed as mean ± SD of at least three independent experiments.

We previously published a series of secondary metabolites from *Neosartorya pseudofischeri*, among which eurochevalierine (Figure 1) displayed interesting anti-cancer properties against multiple cell lines of various origins [28]. More recently, another group further confirmed the relevant cytostatic activity of eurochevalierine in glioblastoma and non-small cell lung cancer cells [29]. Accordingly, in the current study, we sought to further investigate the anti-cancer properties of this sequiterpene alkaloid. Although eurochevalierine displays cytostatic effects in various cancer models, we did not observe eurochevalierine-induced modifications in the cell cycle distribution of cancer cells as revealed by flow cytometry analyses. Nevertheless, a shift of the DNA histograms was observed in eurochevalierine-treated A-549 cancer cells as if treated cells integrated more propidium iodide than control cells (Figure 2A). DNA histogram analyses also revealed that eurochevalierine did not increase the sub-G1 fraction in A-549 lung cancer, U-373 glioblastoma, or B16-F10 melanoma cells, even after 72 h of treatment with a concentration as high as 50 µM (Figure 2B), which nearly corresponds to two times the mean IC_50_ value determined in a panel of cancer cell lines regarding growth inhibition [28]. These results confirmed that eurochevalierine does not behave as a cytotoxic compound and exerts its anti-cancer activity via cytostatic properties.

**Figure 3:**
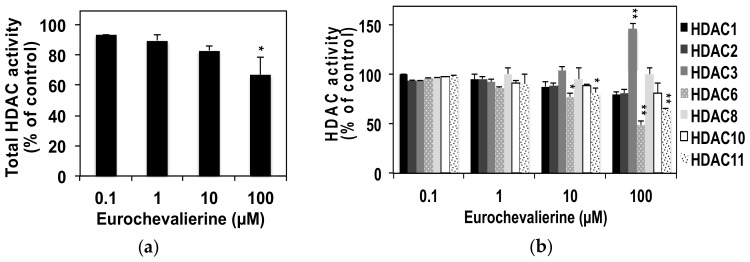
Eurochevalierine displays a moderate effect on in vitro non-sirtuin HDAC activities. (**a**) In vitro total HDAC activity was evaluated using K-562 nuclear extracts in the presence of various concentrations of eurochevalierine. (**b**) Selective in vitro HDAC activity assays were carried out using selected recombinant HDAC proteins in the presence of various concentrations of eurochevalierine. Results are reported as percent of DMSO vehicle control and expressed as the mean ± SD of three independent experiments. * and ** indicate *p* < 0.05 and *p* < 0.01 versus untreated cells, respectively.

Previous studies already revealed that the DNA conformation [30] and modifications in histone H1 concentration [31] influences DNA stainability by propidium iodide as revealed by flow cytometry analyses. It is well established that histone modifications including lysine acetylation and its regulation by HDACs exert major roles in DNA structure [3,26,32]. Thus, we evaluated the effect of eurochevalierine on in vitro HDAC activity. Eurochevalierine inhibited only about 33% of total HDAC activity at a concentration of 100 µM (Figure 3A).

In order to determine whether this effect results from a weak inhibition of multiple HDAC isoenzymes or a strong inhibition of a limited number of selected isoforms, we further tested the effect of eurochevalierine on the deacetylase activity of selected HDAC members. At the concentration of 100 µM, eurochevalierine remained without effect on HDAC1, 2, 8, 10 and 11, inhibited about 36 and 51% of HDAC6 and 11 activities, respectively, and induced HDAC3 activity by 1.5-fold (Figure 3B). Interestingly, eurochevalierine had IC_50_ values of 9.8 and 10 µM against SIRT1 and 2, respectively, whereas it failed to reach the IC_50_ for SIRT3 at 100 µM (Table 1). Such values are comparable to the one obtained with suramin used as a potent positive control for in vitro SIRT1 and SIRT2 inhibition with IC_50_ values of 2.8 and 13 µM, respectively, and was inactive against SIRT3, which is in agreement with previously published studies [33]. The natural SIRT inhibitor nicotinamide was also included as a reference compound for in vitro SIRT3 inhibition [34]. Besides suramin, additional references SIRT1/2, SIRT1, and SIRT2 inhibitors, namely, sirtinol, EX-527, and AGK2, respectively, performed in a similar range or less efficiently [15].

To further explore the potential of eurochevalierine as an SIRT inhibitor, we compared the docking affinity between eurochevalierine and known reference inhibitors (sirtinol, EX-527, and AGK2) using AutoDock Vina [35]. A carboxamide derivative and SirReal2 were used as control docking molecules for SIRT1 and 2, respectively, to validate our docking experiments. The control docking results were very close to the actual binding mode and the overall root-mean-square deviation (RMSD) values between the actual binding mode and simulated docking pose of the carboxamide derivative on SIRT1 and of SirReal2 on SIRT2 were 1.3 Å and 0.4 Å, respectively (Supplementary Materials Figure S1). Qualitative molecular docking experiments were performed with eurochevalierine in comparison with sirtinol and EX-527 against three available human SIRT1 crystal structures that contain two static water molecules in the binding site that would be influential both in the actual inhibition and in the docking experiment [36,37]. The resulting lowest docking affinities are shown in Table 2.

**Figure 4:**
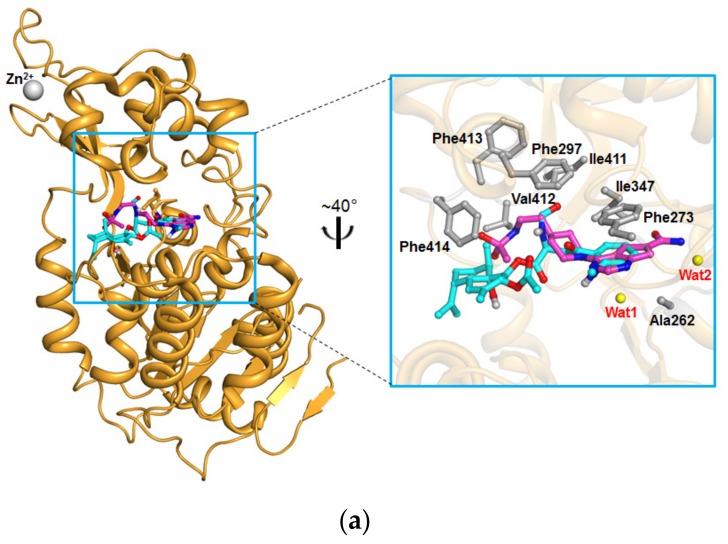

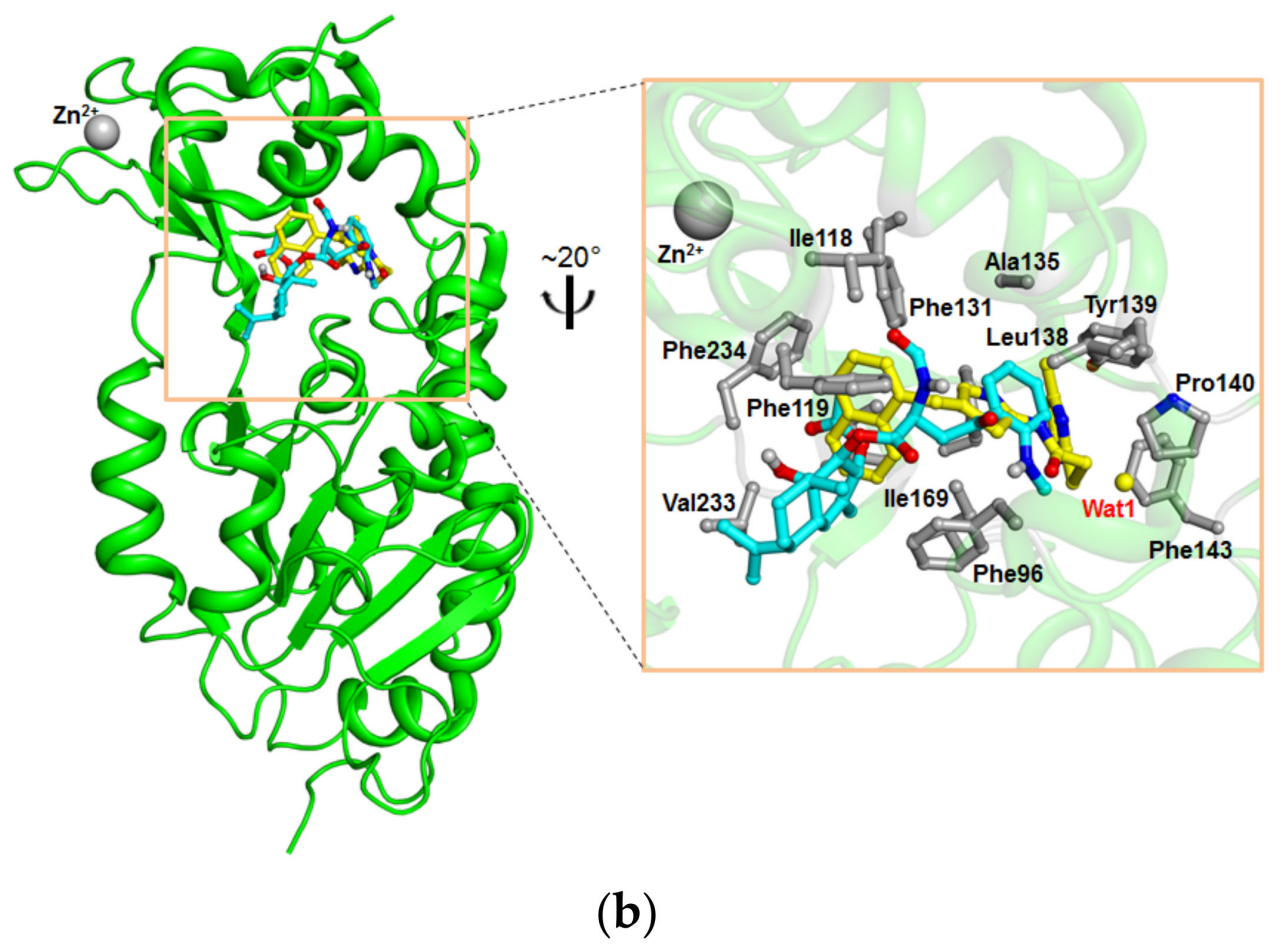
Eurochevalierine docked into human SIRT1 and SIRT2. (**a**) Docking pose of eurochevalierine on the crystal structure of SIRT1 (bright orange; PDB code 4ZZI). The carboxamide derivative (light magenta), the inhibitor in the original SIRT1 crystal structure, was superposed. (**b**) Docking pose of eurochevalierine on the crystal structure of SIRT2 (green; PDB code 4RMH). SirReal2 (yellow), the inhibitor in the original SIRT2 crystal structure, was superposed. Close-up views on the right show that eurochevalierine (cyan) binds to the active site of the SIRT1 and 2 structures. The residues involved in hydrophobic effects with eurochevalierine are represented as gray-colored sticks. Static water molecules are shown as yellow dots.

In Figure 4a, the docking pose of eurochevalierine with the lowest binding affinity was selected as the most favorable binding mode in the binding pocket of SIRT1 [PDB code 4ZZI], and the carboxamide derivative, the inhibitor in the original SIRT1 crystal structure, was superposed for comparison [37]. Even though the binding affinity for eurochevalierine was higher than two reference molecules, sirtinol and EX-527, eurochevalierine was predicted to interact with SIRT1 primarily through hydrophobic effects and to occupy the hydrophobic cage formed by residues Ala262, Phe273, Phe297, Ile347, Ile411, Val412, Phe413, and Phe414, as the control molecule binds to SIRT1. Notably, eurochevalierine exhibits π–π stacks with the side chain of Phe273 like the carboxamide derivative.

In addition, similar docking experiments were performed with eurochevalierine in comparison with sirtinol and AGK2 against three available human SIRT2 crystal structures that contain one static water molecule in the binding site [38,39]. The resulting lowest docking affinities are shown in Table 3. The calculated binding affinity could not correlate our experimental data, such as the IC_50_ values in all aspect, which is mainly caused by the limitation of qualitative docking simulations such as protein flexibility, water/buffer treatments, and the less specific prediction of hydrophobic effects. In Figure 4b, the docking pose of eurochevalierine with the lowest binding affinity was selected as the most favorable binding mode in the binding pocket of SIRT2 (PDB code 4RMH), and SirReal2, the inhibitor in the original SIRT2 crystal structure, was superposed for comparison [38]. Eurochevalierine was predicted to occupy the pocket adjacent to the NAD^+^-binding site like SirReal2 (ref. accession number: 25672491) and to interact with SIRT2 primarily through hydrophobic effects stabilized by the hydrophobic cage (residues Phe96, Ile118, Phe119, Phe131, Ala135, Leu138, Tyr139, Pro140, Phe143, Ile169, Phe190, Ile232, Val233, and Phe234).

**Figure 5:**
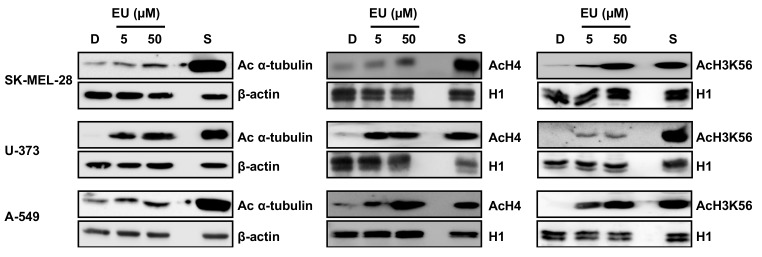
Effect of eurochevalierine on the acetylation status of sirtuin targets. SK-MEL-28, U-373, and A-549 cells were treated with 5 and 50 µM eurochevalierine (EU) for 8 h. Acetylation of histone H4 (AcH4), histone H3 at lysine 56 (AcH3K56), and α-tubulin (Ac α-tubulin) was analyzed via Western blot. β-actin and histone H1 were used as loading controls for the analysis of total and acid extracts, respectively. SAHA (S, 1 µM) was used as a reference HDAC inhibitor. Blots are representative of three independent experiments.

To assess the HDAC inhibitory activity of eurochevalierine in cellulo, we analyzed the acetylation status of SIRT1 and 2 substrates including histone H4, histone H3 at lysine 56 and α-tubulin in SK-MEL-28, U-373, and A-549 cell lines. Since suramin, an SIRT1 and 2 inhibitor reported in the literature, failed to induce any increase in histone or α-tubulin acetylation in our models (Figure S2), cells treated with the pan-HDACi suberoylanilide hydroxamic acid (SAHA) served as a positive control for the induction of protein acetylation. Eurochevalierine induced a robust and dose-dependent increase in the acetylation of sirtuin targets (Figure 5), in agreement with the in vitro inhibition of SIRT1 and 2 activities.

**Figure 6:**
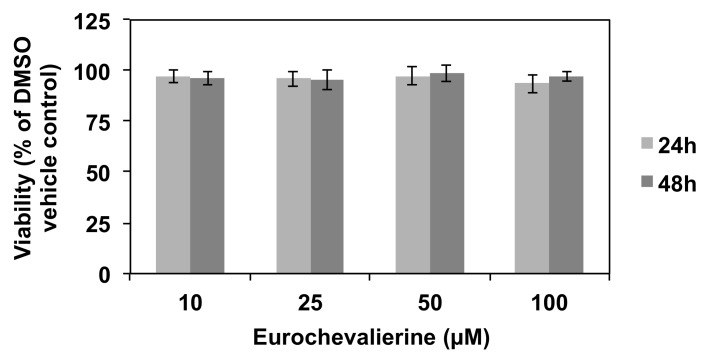
Eurochevalierine does not alter the viability of normal cells. The viability of PBMCs from healthy donors was assessed after 24 and 48 h of treatment at the indicated concentration of eurochevalierine. Results correspond to mean ± SD of three independent experiments.

As a potential candidate for further anti-cancer drug development, we then evaluated the biological activity of eurochevalierine in healthy cell models. First, we tested the effect of eurochevalierine on the viability of peripheral blood mononuclear cells (PBMCs) from healthy donors treated for up to 48 h. Results (Figure 6) revealed that eurochevalierine does not trigger any acute toxicity on this model even at a concentration of 100 µM. 

**Figure 7:**
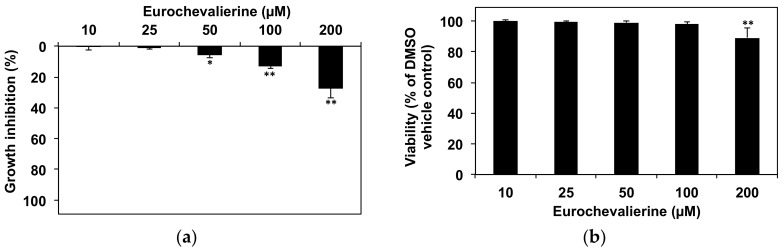
Only very high concentrations of eurochevalierine impair the growth of healthy primary human CD34^+^ stem/progenitor cells. CD34^+^ cells were incubated with the indicated concentrations of eurochevalierine and cell growth (**a**) and viability (**b**) were assessed after 72 h of treatment. Results correspond to the mean ± SD of three independent experiments with * and ** indicate *p* < 0.05 and *p* < 0.01 versus control.

Since eurochevalierine exerts its anti-cancer activity as a cytostatic agent, we further tested the impact of this natural compound on a proliferating healthy cell model represented by primary human CD34^+^ stem/progenitor cells. We observed a maximum growth inhibition of around 30% (Figure 7A) upon 200 µM eurochevalierine even after 72 h of treatment associated with a minor impact on cell viability (Figure 7B). Considering that the mean IC_50_ value for the growth inhibition of cancer cells is 28 µM [28], and that, in health cells, a concentration of 200 µM is not sufficient to reach the IC_50_ (Figure 7A), eurochevalierine displays an important selective toxicity (factor >> 7) for cancer cells compared to normal proliferating cells.

Finally, we wanted to evaluate the drug-likeness properties of eurochevalierine in comparison to other reference SIRTi. Accordingly, we checked whether compounds obey to the extended “rule of five” [molecular weight comprised between 180 and 500 kDa, miLogP ≤ 5, rotatable bonds ≤ 10, topological polar surface area (TPSA) ≤ 140, hydrogen-bond donors ≤ 5 and hydrogen-bond acceptors ≤ 10) combined to an in silico assessment of ADMET (absorption, distribution, metabolism, elimination, and toxicity)] properties (Table 4 and Table S1). 

Collectively, such predictive analyses indicate that eurochevalierine is a suitable drug candidate with more favorable parameters compared with other reference SIRT inhibitors, exhibiting high probability of good oral bioavailability and absorption and a low risk of toxicity. Indeed, the number of rotatable bonds in the eurochevalierine molecule, which defines the molecular flexibility for membrane permeation, was just above the defined limit, and the favorable topological polar surface area, which predicts drug transport properties, is in agreement with a favorable intestinal absorption parameter (92.6%). Unlike eurochevalierine, other compounds such as sirtinol, EX-527, and AGK2 display an unfavorable plasma protein binding potential, which predicts a reduced bio-availability of these compounds. Furthermore, the bio-availability of sirtinol and AGK2 might be further limited due to the high lipophilicity (i.e., high miLogP value). Suramin is a very large molecule with very unfavorable drug-likeness parameters.

## 3. Discussion

It is well accepted that sirtuins play an essential role in cancer cell proliferation and metabolism as well as in aging and inflammation processes [40]. Therefore, modulating sirtuin activities could have therapeutic value. For instance, upregulation of SIRT1 has been reported in multiple cancer cell lines, indicating that SIRT1 inhibitor may be useful as therapeutic agents. Furthermore, non-sirtuin HDACi as well as SIRT2 inhibitors may be of interest to reduce neurodegenerative processes [40]. Here, we identified that the sequiterpene alkaloid eurochevalierine displays a selective sirtuin 1/2 inhibitory profile, which provide a novel chemical scaffold for the lead compound development of SIRT1- and 2-based cancer therapy. Nonetheless, the type of inhibition (i.e., reversible or irreversible) and mechanism (e.g., competitive or noncompetitve) remains to be investigated.

Suramin, originally used for the treatment of trypanosomiasis and onchocerciasis, was described as a potent in vitro SIRTi (SIRT1, 2, and 5) [41]. Accordingly, this symmetric polyanionic naphthylurea compound is frequently used as a positive control for in vitro sirtuin activity assays; however, to the best of our knowledge there is no report attesting to the capacity of suramin to trigger increased lysine acetylation of sirtuin targets. Conversely, we showed in this study that suramin failed to increase the acetylation level of two well characterized sirtuin targets (Figure S2). These results could be explained by the incapacity of suramin to reach in cellulo its cytoplasmic and/or nuclear sirtuin targets due to its relatively high molecular weight or by its inactivation through metabolic transformation. Such hypothesis is in agreement with the unfavorable predicted parameters for suramin drug-likeness (Table 4). In contrast, eurochevalierine was able to inhibit SIRT1 and 2 activities in vitro with IC_50_ values in the same range as suramin but also to induce the acetylation of a cytosolic (α-tubulin) as well as a nuclear target (histone H4) of SIRTs. Furthermore, eurochevalierine appears as a suitable drug candidate with more favorable parameters compared to the other tested reference SIRTi.

Sirtinol [42] as well as cambinol [43] described as selective SIRT1 and 2 inhibitors were previously described to act as potent anti-inflammatory agents. Further study suggested that the anti-cancer properties of sirtinol are linked to decreased proliferation capacities mediated by the development of a senescence-like growth arrest phenotype in human breast cancer MCF-7 and lung cancer H1299 cells [14]. Remarkably, Peck et al. reported that the SIRT1 and 2 inhibitors sirtinol, nicotinamide, and salermide decrease proliferation of cancer cells without affecting cell cycle distribution as observed here with eurochevalierine (Figure 2A), whereas the SIRT1-specific inhibitor EX-527 triggers G1 cell cycle arrest [44]. Similarly, AK-1, a specific SIRT2 inhibitor, induces G1 cell cycle arrest via the upregulation of p21 in colon HT29 cells [45,46]. Recently, it has been shown that EX-527 and AGK2 decrease Hela cervical cancer cell proliferation associated with G1 phase arrest and the downregulation of CDK4 and/or CDK6 [47]. Moreover, effects leading to increased α-tubulin acetylation and modulation of microtubule dynamics and architecture could also contribute to impaired mitosis and proliferation [11]. Altogether these results indicate that the inhibition of SIRT1 and SIRT2 activities by eurochevalierine may account for its cytostatic anti-cancer properties observed previously [28]. 

It should be noted that eurochevalierine also exhibited inhibitory effects against HDAC6 and 11 activities, albeit at higher concentrations. Considering the major role of HDAC6 in regulating many cellular processes and its implication in cancer progression, over the past few years, HDAC6 has emerged as an attractive pharmacological target, whose inhibition alone or in combination treatments displays promising anti-cancer properties in various cancer subtypes including multiple myeloma [8,48]. Thus, we cannot rule out the possibility that these combinational inhibitory effects may contribute to the selective anti-cancer properties of eurochevalierine.

## 4. Materials and Methods

### 4.1. Compounds

Eurochevalierine was isolated from solid culture of *Neosartorya pseudofischeri* according to a procedure previously published [49]. Eurochevalierine, nicotinamide (Sigma, Bornem, Belgium), suramin (Sigma), and SAHA (Cayman, Bio-connect, Huissen, The Netherlands) were dissolved in DMSO.

### 4.2. Cell Culture and Viability Assay

Cancer cell lines were obtained from the American Type Culture Collection (ATCC; Manassas, VA, USA), from the European Collection of Cell Culture (ECACC; Salisbury, UK), and from the Deutsche Sammlung von Mikroorganismen und Zellkulturen (DSMZ; Brauschweig, Germany). These cell lines include the human SK-MEL-28 melanoma (ATCC code HTB-72), the U-373 glioma (ECACC code 08061901), the A-549 non-small-cell lung cancer (DSMZ code ACC107), the K-562 chronic myeloid leukemia (DSMZ code ACC10), and the murine B16-F10 melanoma (ATCC code CRL-6475) models. 

PBMCs were isolated as previously reported [50].

Primary CD34^+^ progenitor/stem cells were isolated from human umbilical cord blood obtained from the Clinique Bohler (Hôpitaux Robert Schuman, Luxembourg, Luxembourg) with the written informed consent of parents with the approval of the National Research Ethics Committee of Luxembourg. First, mononucleated cells were isolated by Ficoll™ (GE Healthcare, Roosendaal, The Netherlands) density gradient centrifugation. CD34^+^ cells were then purified out from mononucleated cells using magnetic cell sorting following the manufacturer’s instructions (MACS Miltenyi, Utrecht, The Netherlands). 

High purity CD34^+^ cells were treated after 3 days of amplification in Stemline^®^ II Hematopoietic Stem cell Expansion Medium (Sigma) supplemented with 1% antibiotic-antimycotic (BioWhittaker^®^, Lonza, Verviers, Belgium), 4 mM l-glutamine (BioWhittaker^®^), 50 ng/mL stem cell factor (ReliaTech, Wolfenbüttel, Germany), and 10 ng/mL interleukin 3 (Reliatech, Wolfenbüttel, Germany). Other cell models were cultured in RPMI1640 (BioWhittaker^®^) supplemented with 10% heat-inactivated fetal calf serum (BioWhittaker^®^) and 1% antibiotic-antimycotic. Cell lines were cultured at 37 °C in a humid atmosphere and at 5% CO_2_.

PBMCs and CD34^+^ cells were processed through a semi-automated image-based cell analyzer (Cedex XS Innovatis, Roche, Luxembourg), which provides information about cell concentration and viability based on the Trypan Blue exclusion method.

### 4.3. Flow Cytometry

Cells were collected, fixed, and stained with the APO AF TUNEL detection kit (BD, Erembodegem, Belgium) allowing analysis of both apoptosis and cell cycle at the same time [28]. The experiment was conducted accordingly to the procedure of the manufacturer. Data were obtained through flow cytometry with a Cell Lab Quanta apparatus (Analis, Suarlee, Belgium).

### 4.4. In Vitro HDAC Activity Assay

HDAC assays were carried out as previously described [51].

### 4.5. Docking Studies

Docking studies were performed as previously described [15] with a docking grid size of 25 Å × 25 Å × 25 Å, which encompassed the entire inhibitor binding pocket of SIRT1 and 2 structures. To prepare the SIRT coordinates for docking simulation, ligands and all water molecules were removed except static water molecules in the binding site that could play important roles in the actual inhibition.

### 4.6. Protein Extraction and Western Blotting

Total proteins were extracted using the MPER^®^ reagent (Thermo-scientific, Erembodegem-Aalst, Belgium) supplemented by 1× protease inhibitor cocktail (Complete EDTA-free, Roche, Prophac, Luxembourg, Luxembourg) according to manufacturer’s instructions. Histones were extracted as previously described [52]. Western blots were carried out as previously reported [53]. 

Anti-acetylated α-tubulin (sc-23950) was from Santa Cruz Biotechnology (Tebubio, Boechout, Belgium); anti-acetylated histone H4 (06-866), anti-acetylated histone H3 lysine 56 (04-1135), anti-histone H1 (05-457) were from Millipore (Brussels, Belgium); anti-β-actin (A5441) was from Sigma. Corresponding secondary antibodies were from Santa Cruz Biotechnology.

### 4.7. Calculation of Drug-Like Properties

Important molecular properties to predict the drug-likeness of a compound were computed using the web-based Molinspiration [54] and PreADMET ver 2.0 [55] programs. 

### 4.8. Statistics

Statistical analyses were carried out using the GraphPad Prism 6.0 software (GraphPad Software, La Jolla, CA, USA). One-way ANOVA followed by the Holm–Sidak multiple comparison tests were used for statistical comparisons. *p*-values less than 0.05 were considered statistically significant. 

## Figures and Tables

**Table 1:** In vitro inhibitory activity of selected inhibitors and eurochevalierine on SIRT1, -2, and -3 activities.

Compound	IC_50_ (µM) ^1^		
	SIRT1	SIRT2	SIRT3
Nicotinamide ^2^	97 ± 15	27 ± 3	67 ± 10
Suramin ^3^	2.8 ± 0.3	13 ± 1	>100 ^4^
Sirtinol ^3^	82.5 ± 7.1	47.1 ± 4.0	ND
EX-527 ^3^	0.10 ± 0.06	20.1 ± 4.2	ND
AGK2 ^3^	98.1 ± 2.4	2.8 ± 1.0	ND
Eurochevalierine	9.8 ± 2.0	10.2 ± 3.9	>100 ^5^

^1^ IC_50_ values represent the mean ± SD of three independent experiments. ND: not determined. ^2^ Nicotinamide was use as in vitro reference SIRT3 inhibitor. ^3^ Suramin and sirtinol, EX-527, and AGK2 were used as in vitro reference inhibitors of SIRT1/2, SIRT1, and SIRT2, respectively. Data from [15] obtained in the same experimental conditions. ^4^ 70% and ^5^ 59% are the remaining enzymatic activity at 100 µM compared to the control set to 100%.

**Table 2:** Qualitative molecular docking results of eurochevalierine against human SIRT1 ^1^.

PDB_ID	Eurochevalierine	Sirtinol	EX-527
4I5I	−8.0	−9.0	−10.3
4ZZI	−9.0	−10.8	−8.6
4ZZJ	−9.0	−9.7	−8.9
Average	−8.7	−9.8	−9.3

^1^ Binding affinity energy values (kcal/mol) with SIRT1 proteins in the indicated protein data bank (PDB) codes. Sirtinol and EX-527 were used as reference inhibitors of SIRT1/2 and SIRT1, respectively.

**Table 3:** Qualitative molecular docking results of eurochevalierine against human SIRT2 ^1^.

PDB_ID	Eurochevalierine	Sirtinol	AGK2
4RMG	−9.0	−10.2	−11.2
4RMH	−10.0	−9.1	−11.8
5DY4	−9.1	−10.4	−10.9
Average	−9.4	−9.9	−11.3

^1^ Binding affinity energy values (kcal/mol) with SIRT2 proteins in the indicated PDB codes. Sirtinol and AGK2 were used as reference inhibitors of SIRT1/2 and SIRT2, respectively.

**Table 4:** In silico drug-likeness parameters calculated for reference sirtuin inhibitors (SIRTi) and eurochevalierine.

Method	Parameter ^1^	Values						
		Theoretical	Eu	Suramin	Nicotinamide	Sirtinol	EX-527	AGK2
Rule of 5	n-atoms	20 ≤ *x* ≤ 70	38	86	9	30	17	30
	MW (KDa)	180 ≤ *x* ≤ 500	526.63	1297.3	122.13	394.47	248.71	434.28
	miLogP	≤5	4.04	−5.72	−0.48	5.67	2.51	5.73
	TPSA	≤140	131.03	483.74	55.99	61.69	58.88	78.92
	n-ON	≤10	9	29	3	4	3	5
	n-OHNH	≤5	3	12	2	2	3	1
	n-rotb	≤10	11	16	1	5	1	4
Absorption	BBBP	0.1 ≤ MA ≤ 2	0.10	0.04	0.34	3.10	4.10	0.08
	IA	≥70%	92.6	65.2	93	95.8	90.3	97.3
	PPB	<90%	84.8	100	2.03	91.1	91.0	97.5
Toxicity	Rat	NA	Negative	Negative	Negative	Negative	Negative	Positive
	Cardiac	NA	Ambigous	Ambigous	Medium	Ambigous	Medium	Medium

^1^ BBBP: blood-brain barrier penetration; IA: intestinal absorption; miLogP: octanol-water partition coefficient; MW: molecular weight; n-atoms: number of atoms; n-OHNH: number of hydrogen bond donors; n-ON: number of hydrogen acceptors; n-rotb: number of rotatable bonds; PPB: plasma protein binding; TPSA: topological polar surface area. EU: eurochevalierine; MA: middle absorption; NA: not applicable.

## Supplementary Materials

The following are available online; Figure S1: Control docking results; Figure S2: Suramin fails to induce histone H4 or α-tubulin acetylation in K-562 cells; Table S1: Global drug-likeness parameters calculated for reference sirtuin inhibitors and eurochevalierine.
