# Supplementary material for: The Fungal Metabolite Eurochevalierine, a Sequiterpene Alkaloid, Displays Anti-Cancer Properties through Selective Sirtuin 1/2 Inhibition
Source: Molecules. 2018 Feb 5;23(2):333. doi: 10.3390/molecules23020333 (PMC6017873; doi:10.3390/molecules23020333)
Supplement: Supplementary file 1 [file molecules-23-00333-s001.zip › 1/molecules-266867-supplementary-revised/Schnekenburger et al_Supplementary/Schnekenburger et al-suppl figure 2.pdf]

Fig.S1

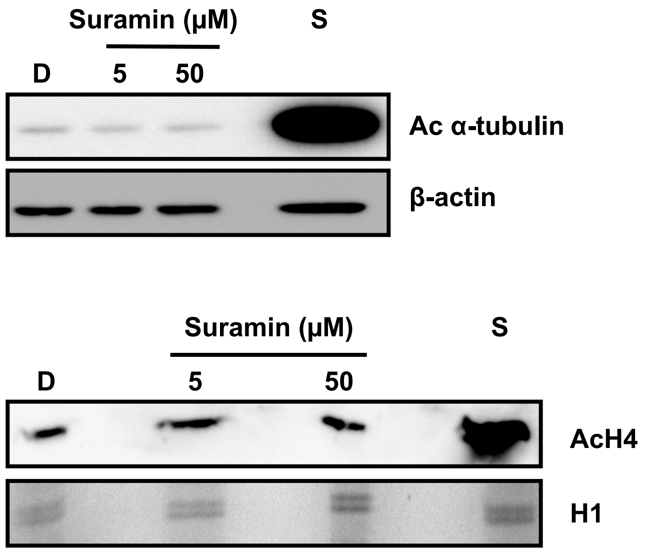

Figure S2. Suramin fails to induce histone H4 or α-tubulin acetylation in K-562 cells. K-562 cells were treated with DMSO (D), 5 or 50 µM eurochevalierine and SAHA (S; 1 µM). Acetylation of histone H4 (AcH4) and α-tubulin (Ac α-tubulin) was analyzed by Western Blot. β-actin and histone H1 were used as loading controls for the analysis of total and acid extracts, respectively. Blots are representative of three independent experiments.
