# Supplementary material for: The Fungal Metabolite Eurochevalierine, a Sequiterpene Alkaloid, Displays Anti-Cancer Properties through Selective Sirtuin 1/2 Inhibition
Source: Molecules. 2018 Feb 5;23(2):333. doi: 10.3390/molecules23020333 (PMC6017873; doi:10.3390/molecules23020333)
Supplement: Supplementary file 1 [file molecules-23-00333-s001.zip › 1/molecules-266867-supplementary-revised/Schnekenburger et al_Supplementary/Schnekenburger et al_Table S1.pdf]

Table S1: Global drug-likeness parameters calculated for reference sirtuin inhibitors and eurochevalierine.

Method	Parameter	Compound					
		**Eurochevalierine**	**Suramin**	**Nicotinamide**	**Sirtinol**	**EX-527**	**AGK2**
Molinspiration	miLogP	4.04	-5.72	-0.48	5.67	2.51	5.73
	TPSA		483.74	55.99	61.69	58.88	78.92
	natoms	38	86	9	30	17	30
	MW	526.63	1297.3	122.13	394.47	248.71	434.28
	nON	9	29	3	4	3	5
	nOHNH	3	12	2	2	3	1
	nviolations	1	3	0	1	0	1
	nrotb	11	16	1	5	1	4
	volume	494.41	968.22	110.16	366.9	213.22	351
Druglikeness	CMC_like_Rule	Not qualified	Not qualified	Not qualified	Qualified	Qualified	Qualified
	CMC_like_Rule_Violation_Fields	Molecular_weight, AMolRef,	Molecular_weight, AMolRef,	Molecular_weight, AMolRef,			
	CMC_like_Rule_Violations	3	3	3	0	0	0
	Lead-like_Rule_Violation_Fields	Molecular_weight	Molecular_weight, AlopP98_value	AlopP98_value	Molecular_weight, AlopP98_value	AlopP98_value	Molecular_weight, AlopP98_value
	Lead_like_Rule	Violated	Violated	Violated	Violated	Violated	Violated
	Lead_like_Rule_Violations	1	2	1	2	1	2
	MDDR_like_Rule	Drug-like	Mid-structure	Nondrug-like	Mid-structure	Mid-structure	Mid-structure
	MDDR_like_Rule_Violation_Fields		No_Rotatable_bonds	No_Rings, No_Rigid_bonds,	No_Rotatable_bonds	No_Rotatable_bonds	No_Rotatable_bonds
	MDDR_like_Rule_Violations	0	1	3	1	1	1
	Rule_of_Five	Suitable	Violated	Suitable	Suitable	Suitable	Suitable
	Rule_of_Five_Violation_Fields	Molecular_weight	Molecular_weight, No_H_bond_acceptors,		AlopP98_value		AlopP98_value
	Rule_of_Five_Violations	1	3	0	1	0	1
	WDI_like_Rule	Out of 90% cutoff	Out of 90% cutoff	Out of 90% cutoff	Out of 90% cutoff	In 90% cutoff	Out of 90% cutoff
	WDI_like_Rule_Violation_Fields	AMolRef, Kier_flexibility, Kier_alpha_01, Kier_alpha_02, VChi_00, VChi_01, VChi_02, Wiener_index, 1st_Zagreb	No_H_bond_acceptors, No_H_bond_donors, AMolRef, Kier_flexibility, Kier_alpha_01, Kier_alpha_02, Kier_alpha_03,	Balaban_index_JX	AlopP98_value, AMolRef		AlopP98_value
	WDI_like_Rule_Violations	9	15	1	2	0	1
ADME	BBB	0.0988756	0.0355754*	0.337286	3.10148	4.10369	0.0836373
	Buffer_solubility_mg_L	0.00201616	80.8068**	2796.2	0.045153	321.867	59.6871**
	Caco2	21.4869	0.831496	20.3605	28.4191	21.3007	26.6938
	CYP_2C19_inhibition	Non	Non	Inhibitor	Non	Inhibitor	Non
	CYP_2C9_inhibition	Non	Inhibitor	Inhibitor	Inhibitor	Inhibitor	Non
	CYP_2D6_inhibition	Non	Non	Inhibitor	Non	Non	Non
	CYP_2D6_substrate	Non	Non	Non	Non	Non	Non
	CYP_3A4_inhibition	Inhibitor	Inhibitor	Inhibitor	Non	Inhibitor	Non
	CYP_3A4_substrate	Substrate	Substrate	Non	Weakly	Non	Weakly
	HIA	92.606444	65.212934	92.948174	95.772797	90.330406	97.265034
	MDCK	0.0447817	0.0180481*	5.70167	0.0726271	50.9482	0.0919261
	Pgp_inhibition	Non	Inhibitor	Non	Inhibitor	Non	Inhibitor
	Plasma_Protein_Binding	84.822896	100	2.029501	91.126218	90.949028	97.529775
	Pure_water_solubility_mg_L	0.222789	2.71E-07	78993	0.0581602	22.5921	0.0152093
	Skin_Permeability	-3.09425	-0.790679*	-3.71578	-2.02133	-4.31139	-2.46653
	SKlogD_value	3.22105	-3.74189	-0.25836	5.23865	2.79476	5.50681
	SKlogP_value	3.22105	2.43673	-0.25836	5.23865	2.79476	5.50681
	SKlogS_buffer	-8.41698	-4.205580**	-1.64024	-6.94133	-2.88802	-3.861890**
	SKlogS_pure	-6.37361	-12.67952	-0.18922	-6.83139	-4.04174	-7.45566
Toxicity	algae_at	0.00715077	3.10E-06	0.435369	0.00764099	0.03319	0.00448356
	Ames_test	mutagen	non-mutagen	mutagen	mutagen	mutagen	mutagen
	Carcino_Mouse	positive	positive	positive	positive	positive	negative
	Carcino_Rat	negative	negative	negative	negative	negative	positive
	daphnia_at	0.0469835	9.53E-05	3.47006	0.0067202	0.0607803	0.00251836
	hERG_inhibition	ambiguous	ambiguous	medium_risk	ambiguous	medium_risk	medium_risk
	medaka_at	0.00526705	2.67E-07	12.0931	0.000123046	0.00654108	2.17E-05
	minnow_at	0.00417433	3.45E-07	3.01776	0.000452177	0.00929953	8.68E-05
	TA100_10RLI	negative	negative	positive	negative	positive	negative
	TA100_NA	negative	negative	positive	negative	negative	positive
	TA1535_10RLI	negative	negative	positive	negative	negative	negative
	TA1535_NA	negative	negative	positive	negative	positive	negative
