# Supplementary material for: The Fungal Metabolite Eurochevalierine, a Sequiterpene Alkaloid, Displays Anti-Cancer Properties through Selective Sirtuin 1/2 Inhibition
Source: Molecules. 2018 Feb 5;23(2):333. doi: 10.3390/molecules23020333 (PMC6017873; doi:10.3390/molecules23020333)
Supplement: Supplementary file 1 [file molecules-23-00333-s001.zip › 1/molecules-266867-supplementary-revised/Schnekenburger et al_Supplementary/Schnekenburger_Suppl figure 1.pdf]

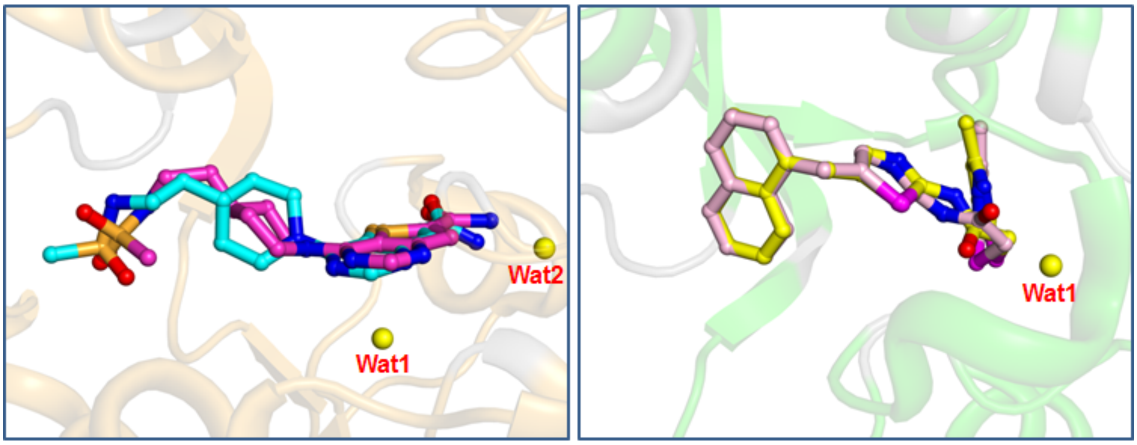


**Figure S1.** Control docking results (a) Superposition of the actual binding mode (light magenta) and the simulated docking pose (cyan) of the carboxamide derivative on the crystal structure of SIRT1 (PDB code 4ZZI) with RMSD of 1.3 Å. (b) Superposition of the actual binding mode (yellow) and the simulated docking pose (light pink) of SirReal2 on the crystal structure of SIRT2 (PDB code 4RMH) with RMSD of 0.4 Å. Static water molecules are represented as yellow dots.
