# Supplementary figures and images for: The Fungal Metabolite Eurochevalierine, a Sequiterpene Alkaloid, Displays Anti-Cancer Properties through Selective Sirtuin 1/2 Inhibition
Source: Molecules. 2018 Feb 5;23(2):333. doi: 10.3390/molecules23020333 (PMC6017873; doi:10.3390/molecules23020333)

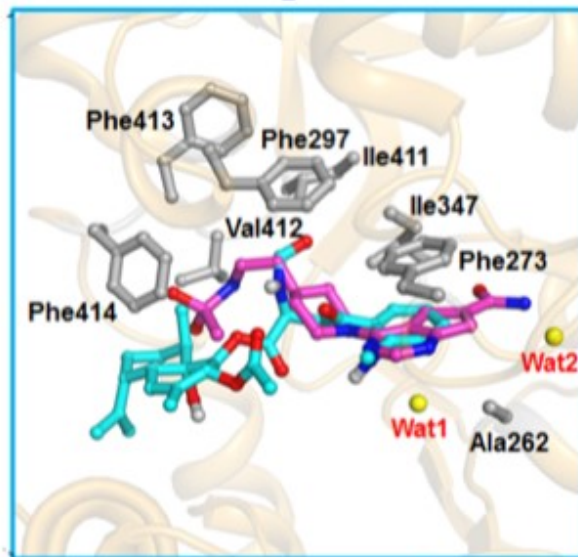

**SIRT1**

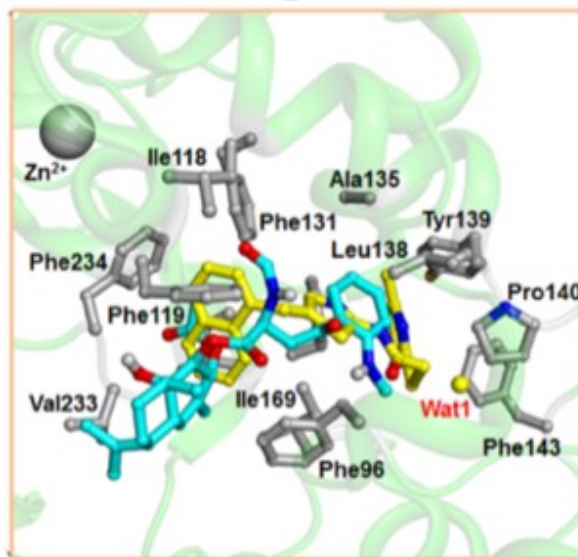

**SIRT2**

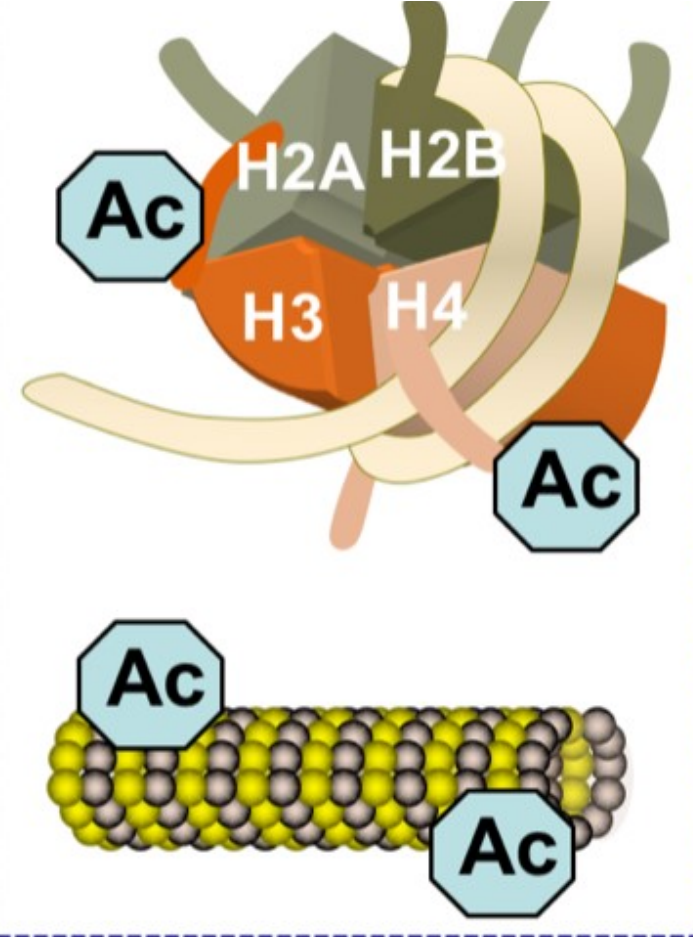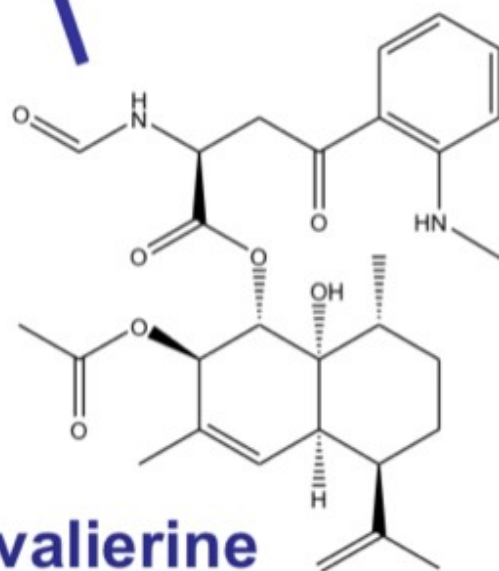

**Eurochevalierine**  
(from *Neosartorya pseudofischeri*)

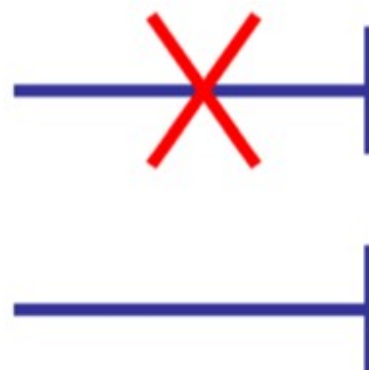

**Healthy cells**

**Cancer cells**

Supplement: Supplementary file 1 [file molecules-23-00333-s001.zip › 1/molecules-266867-graphic.pdf]
